# The role of smoking in the relationship between intimate partner violence and age at natural menopause: a mediation analysis

**DOI:** 10.1186/s40695-017-0031-9

**Published:** 2018-01-15

**Authors:** Gita D. Mishra, Hsin-Fang Chung, Yalamzewod Assefa Gelaw, Deborah Loxton

**Affiliations:** 1 School of Public Health, The University of Queensland, Herston Road, Herston, Brisbane, QLD 4006 Australia; 2 Research Centre for Generational Health and Ageing, The University of Newcastle, Callaghan, NSW Australia

**Keywords:** Age at natural menopause, Intimate partner violence, Mediation, Smoking

## Abstract

**Background:**

Age at natural menopause (ANM) is considered as a biologic marker of health and ageing. The relationship between intimate partner violence (IPV) and ANM is currently unknown, and whether smoking plays a role in this relationship is unclear. The aim of this study was to examine the association between IPV and ANM and to quantify the effect mediated through smoking.

**Methods:**

Data were drawn from the 1946–51 cohort of the Australian Longitudinal Study on Women’s Health, a prospective cohort study first conducted in 1996. History of IPV (yes or no) was self-reported at baseline. ANM was confirmed by at least 12 months of cessation of menses where this was not a result of medical interventions such as bilateral oophorectomy or hysterectomy and categorised as <45 (early menopause), 45–49, 50–51, 52–53, and ≥54 years. Regression models and mediation analyses based on the counterfactual framework were performed to examine the relationship between IPV and ANM and to quantify the proportion mediated through smoking (never, past, current <10, 10–19 and ≥20 cigarettes/day).

**Results:**

Of 6138 women in the study with natural menopause, 932 (15%) reported a history of IPV and 429 (7.0%) had an early ANM (before age 45 years). Women with IPV were more likely to smoke and be heavy smokers (Odds Ratio: 2.77, 95% CI 2.19–3.51). Women with IPV were also at increased risk of early menopause (ANM <45 years) (Relative Risk Ratio: 1.36, 95% CI 1.03–1.80) after accounting for education level, income difficulties, age at menarche, parity, body mass index, and perceived stress, compared to the reference group (women without IPV and ANM at 50–51 years). This relationship was attenuated after adjusting for smoking (Relative Risk Ratio: 1.20, 95% CI 0.90–1.59). Mediation analysis showed that cigarette smoking explained 36.7% of the association between IPV and early menopause (ANM <45 vs. ≥45 years).

**Conclusion:**

Cigarette smoking substantially mediated the relationship between IPV and early menopause. Findings suggest that as part of addressing the impact of IPV, timely interventions that result in cessation of smoking will partly mitigate the increased risk of early menopause.

**Electronic supplementary material:**

The online version of this article (10.1186/s40695-017-0031-9) contains supplementary material, which is available to authorized users.

## Background

While the exact definition of intimate partner violence (IPV) varies between countries, the World Health Organization (WHO) defines IPV as physical violence, sexual violence, stalking and psychological aggression (including coercive acts) by a current or former intimate partner [1]. In 2013, the WHO multi-country study report documented the global prevalence of physical and/or sexual IPV was 30.0% (95% CI 27.8–32.2%), with the highest levels (approximately 37%) reported in the WHO African, Eastern Mediterranean and South-East Asia Regions [2, 3]. However, IPV against women can occur in all settings, age and socioeconomic groups [4] and is increasingly recognised as a pattern of behaviour that has both immediate and long term consequences for health and well-being [2, 5, 6]. The impact related to reproductive health for women who have experienced some form of IPV includes unintended and/or unwanted pregnancy, abortion, sexually transmitted diseases, cervical cancer and vaginal discharge [2, 6, 7]. The prevalence of smoking is also more likely to be higher among women who have experienced IPV [8–10].

Findings from Australia suggests that IPV is responsible for 8% of overall disease burden for women discussed as selected risks to health [11, 12]. An 11-year population-based study of mid-aged Australian-born women showed IPV was significantly associated with poorer mental and sexual health status [13], and analysis of data from the Australian Longitudinal Study on Women’s Health (ALSWH) demonstrated mental and physical health deficits attributable to IPV that lasted the length of the 16 year study period [14]. A study in Victoria measuring the impact of IPV on the health of women reported that IPV accounted for 2.9% of the total disease and burden for women of all ages [15].

The age at natural menopause (ANM), which marks the cessation of menses, acts as a biomarker for reproductive ageing. The timing of ANM is also linked to a range of cardiovascular and metabolic conditions in later life [16], such as earlier ANM and increased risk of ischemic stroke [17]. While a range of factors is linked to the timing of ANM, smoking is one of the most well-established risk factors for earlier menopause [18]. Our earlier cross-sectional analysis of baseline ALSWH data indicated that smoking and postmenopausal status were associated with IPV among women aged 45–50 years [7]. However, 41% of women in the sample had not yet reached menopause, and the nature of the data precluded longitudinal analysis. Furthermore, the underlying mechanism by which IPV might act to drive early menopause was not identified. To date, there have been no studies that clearly identify links between IPV and ANM. Given that cigarette smoking is associated with both IPV and ANM, the aim of this study is to examine if IPV is associated with ANM and to investigate the role of cigarette smoking as a potential mediator of ANM by using a counterfactual framework for mediation analyses.

## Methods

### Study design and population

The Australian Longitudinal Study on Women’s Health (ALSWH) is an ongoing population-based cohort study of factors affecting the health and well-being of Australian women born in 1921–26, 1946–51, and 1973–78. Women were randomly selected from the national Medicare dataset, which covers all citizens and permanent residents of Australia. Women were first surveyed in 1996 and were followed every 2–4 years using self-completed questionnaires. Full details of the study design, recruitment and response rates have been reported elsewhere [19, 20]. The study protocols were approved by the Human Research Ethics Committees of the University of Newcastle and University of Queensland, Australia. Informed consent was obtained from all participants at each survey.

The present study focused on the 1946–51 cohort, which was first surveyed in 1996 when the women were aged 45 to 50 years (Survey 1, *n* = 13,714), and then in 1998 (Survey 2, *n* = 12,338), 2001 (Survey 3, *n* = 11,226), 2004 (Survey 4, *n* = 10,905), 2007 (Survey 5, *n* = 10,638), 2010 (Survey 6, *n* = 10,011) and 2013 (Survey 7, *n* = 9151). ANM was determined for 7635 women who reported to have natural menopause (not a result of medical interventions) and recorded their age at menopause over the study period. Among these women, 1497 were excluded due to missing baseline data on history of IPV (*n* = 44), smoking status (*n* = 236) and relevant covariates including education level (*n* = 46), income difficulties (*n* = 36), body mass index (BMI) (*n* = 223), perceived stress (*n* = 26), number of children (*n* = 179), and age at menarche (*n* = 707). Therefore, data from 6138 women were included in the analyses.

### Main outcome and exposure variables

Age at menopause was determined from responses to the question “if you have reached menopause, at what age did your periods completely stop?” asked in Survey 2 to Survey 6. ANM was confirmed by at least 12 months of cessation of menses where this was not a result of medical interventions such as surgical menopause due to bilateral oophorectomy or hysterectomy. If the ANM was reported at multiple surveys, data reported at the last available survey were used. ANM was treated as a continuous variable and was categorised as <45 (early menopause), 45–49, 50–51, 52–53 and ≥54 years [21].

IPV was defined from responses to the question at baseline “have you ever been in a violent relationship with a partner/spouse?” and categorised women as with or without a history of IPV. Women were also asked a question at Survey 5 “if you have ever lived with a violent partner or spouse, in which years did you experience violence?” Nearly 90% of the women reported they had experienced IPV before 1996 (baseline), which would indicate that the majority of victims had their first IPV experience before midlife.

### Smoking and covariates

Smoking status was reported at baseline and categorised as never, past smoker, and current smoker with <10, 10–19 and ≥20 cigarettes per day. Other baseline covariates included area of residence (categorised as urban and rural/remote), education level (no formal qualifications, less than high school/high school, trade/certificate/diploma and university or higher), difficulty on income management (easy/not bad/some difficult and difficult/impossible), marital status (married/de facto, separated/divorced, widowed and single), age at menarche (≤11, 12, 13, 14 and ≥15 years) and number of children (parity) (0, 1, 2–3 and ≥4 children). BMI was computed as self-reported weight (kg) divided by the square of height (m) and categorised as underweight (<18.5 kg/m^2^), normal weight (18.5–24.9 kg/m^2^), overweight (25–29.9 kg/m^2^) and obese (≥30 kg/m^2^). Perceived stress levels at baseline were assessed by asking participants to rate how stressed they had been in the last 12 months for the following life domains: own health, health of other family members, work/employment, living arrangements, study, money, relationship with parents, relationship with partner/spouse, relationship with children, relationship with other family members. The performance of this preceived stress scale was demonstrated with internal reliability and construct validity [22, 23]. The range of summary stress scores was from 0 to 4. Higher scores indicate more perceived stress. The scores were categorised as not at all stressed (0), somewhat stressed (<1), moderately stressed (1 to <2), very stressed (2 to <3) and extremely stressed (3 to 4). In our analysis, we dichotomised stressed status as absence (scores <1) and presence (scores ≥1).

### Statistical analysis

Participant characteristics at baseline were described according to the history of IPV (yes or no) and five categories of ANM (<45, 45–49, 50–51, 52–53 and ≥54 years). Descriptive statistics were presented as percentages for categorical data and the median (interquartile range) for continuous data. Chi-squared tests and regression models were used to examine the differences between groups.

**Fig. 1:**
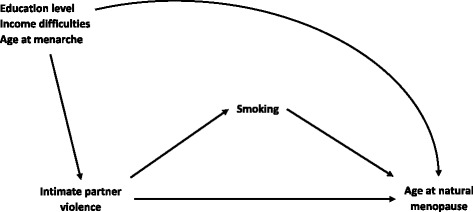
Directed acyclic graph for mediation analysis of the relationships between intimate partner violence (exposure), smoking (mediator) and age at natural menopause (outcome)

To examine the contribution of smoking to the IPV and ANM relationship, the causal diagram presented in Fig. 1 was formulated. IPV was hypothesised to affect ANM both directly and indirectly, in which smoking acted as a mediator. Interactions between IPV and smoking categories on ANM were tested and taken into account if significant. Education level, income difficulties, and age at menarche were the background confounders. High parity, obesity, and stress could be the consequence of IPV [24, 25], thus they were not considered as confounders in the causal diagram. To test these hypotheses, two complementary approaches were used. First, a series of logistic and linear regression models were performed to examine the relationship between IPV and ANM. Multinomial logistic regression models with five categories of outcome for ANM were used to estimate relative risk ratio (RRR) and 95% confidence interval (CI) with age 50–51 as the reference. Linear regression models were used to examine the association with ANM as a continuous outcome. Sequential multivariable regression models were built following multiple adjustment plans by adjusting for education level (Model 1), income difficulties (Model 2), age at menarche (Model 3), parity (Model 4), BMI (Model 5), stress status (Model 6), and subsequently further adjusting for smoking status (Model 7). Attenuated associations between IPV and ANM were expected after adjustment for smoking status, which would indicate a potential mediating role of smoking. Factors that were associated with IPV but did not affect the association between IPV and ANM included area of residence and marital status, thus they were not included in our models.

Second, we performed a formal mediation analysis by using the counterfactual approach [26, 27]. Using the counterfactual framework allows for decomposition of the total effect of IPV on ANM into natural direct and indirect effects mediated through smoking, even in models with non-linearities (e.g. when ANM and smoking are considered as a binary variable) and interactions (e.g. when the effect of IPV is worsened by smoking) [26, 27]. The mediation analysis was performed by fitting a logistic regression model for the binary outcome (ANM <45 and ≥45 years) and a linear regression model for ANM as a continuous outcome; fitting a linear or logistic regression model for the continuous mediator (treating the ordinal variable for smoking status as continuous) or binary mediator (dicotomised as current and non-current smoking) [28]. Models were adjusted for education level, income difficulties, and age at menarche. Interactions between IPV and smoking were not statistically significant from our preliminary regression analyses (p for interaction >0.4), thus no exposure-mediator interaction was included in our models. From these combined models, we obtained odds ratios (ORs) of natural direct effect (OR^NDE^), natural indirect effect (OR^NIE^) and total effect (OR^TE^) for the binary outcome, while for the linear outcome, we derived parameter estimates of β^NDE^, β^NIE^ and β^TE^. The proportion mediated through mediator was calculated on the risk difference scale. The proportion mediated was calculated as [OR^NDE^ (OR^NIE^ – 1)] / [OR^NDE^ × OR^NIE^ –1] × 100% for the binary outcome (assuming the outcome of early menopause <45 years is relatively rare) [29] or calculated as (β^NIE^/β^TE^) × 100% for the continuous outcome.

Given that women who were excluded from the analysis due to incomplete data were more likely to be less educated, be stressed, be a current smoker, and have a history of IPV, we performed a sensitivity analysis using inverse probability weighting to account for this bias [30]. Logistic regression model was used to calculate propensity scores as the predicted probability of having data observed (i.e. not being missing). We performed a complete-case analysis but weighted the complete cases by the inverse of their probability of being a complete case. Statistical analyses were performed using SAS 9.4 (SAS Institute, Inc., Cary, North Carolina). The paramed program in STATA 14 (StataCorp LP, College Station, Texas) was used to perform the mediation analyses and estimate total, natural direct and natural indirect effects [28]. A 2-sided *p* < 0.05 was considered statistically significant.

## Results

This study included 6138 women experiencing natural menopause. Compared with these women, those who were excluded due to incomplete data were more likely to have a lower level of education, have income difficulties, have a history of IPV, be stressed, be a current smoker and have an early ANM (Additional file 1: Table S1). For women included in the study, the mean ANM was 50.9 years (median 51.0 years, interquartile range: 49.0–54.0), with 7% of the women having early menopause (<45 years).

At baseline (when aged 45–50 years), almost one in six women (15%) reported that they had experienced IPV (Table 1). After adjusting for other factors, women with a history of IPV were more likely to live in rural/remote areas, to have lower education levels, to have income difficulties, to be single or separated/divorced, to have four or more children, and to be obese than women without IPV. Women who had suffered IPV were more than twice as likely to report being stressed (OR 2.03, 95% CI 1.72–2.40) or be current smokers, with a markedly higher likelihood of being a heavy smoker (20 or more cigarettes per day) (OR 2.77, 2.19–3.51). This was also evident in the higher prevalence of heavy smokers (17%) among women with a history of IPV, compared with other women (7%).

**Table 1:** Baseline characteristics of women according to their history of intimate partner violence (*n* = 6138)

	History of intimate partner violence (IPV)			
	No (*n* = 5206, 84.8%)	Yes (*n* = 932, 15.2%)	Crude OR (95% CI)	Adjusted^a^ OR (95% CI)
Area of residence				
Urban	1913 (36.7)	307 (32.9)	Reference	Reference
Rural/remote	3293 (63.3)	625 (67.1)	1.18 (1.02–1.37)	1.25 (1.06–1.47)
Education level				
No formal qualifications	666 (12.8)	168 (18.0)	1.90 (1.48–2.44)	1.74 (1.32–2.30)
Less than high school/high school	2497 (48.0)	468 (50.2)	1.41 (1.14–1.74)	1.45 (1.15–1.82)
Trade/certificate/diploma	1102 (21.2)	171 (18.3)	1.17 (0.91–1.49)	1.21 (0.93–1.58)
University or higher	941 (18.1)	125 (13.4)	Reference	Reference
Difficulty on income management				
Easy/not bad/some difficult	4685 (90.0)	701 (75.2)	Reference	Reference
Difficult/impossible	521 (10.0)	231 (24.8)	2.96 (2.49–3.53)	1.66 (1.36–2.02)
Marital status				
Married/de facto	4548 (87.5)	606 (65.1)	Reference	Reference
Separated/divorced	399 (7.7)	272 (29.2)	5.12 (4.29–6.10)	4.29 (3.55–5.20)
Widowed	97 (1.9)	18 (1.9)	1.39 (0.84–2.32)	1.30 (0.76–2.23)
Single	156 (3.0)	35 (3.8)	1.68 (1.16–2.45)	2.27 (1.45–3.57)
Age at menarche (years)				
≤ 11	883 (17.0)	184 (19.7)	1.23 (1.00–1.52)	1.10 (0.88–1.38)
12	1109 (21.3)	178 (19.1)	0.95 (0.77–1.17)	0.92 (0.74–1.15)
13	1526 (29.3)	258 (27.7)	Reference	Reference
14	899 (17.3)	144 (15.5)	0.95 (0.76–1.18)	0.94 (0.74–1.19)
≥ 15	789 (15.2)	168 (18.0)	1.26 (1.02–1.56)	1.18 (0.94–1.48)
Number of children				
0	458 (8.8)	54 (5.8)	0.74 (0.55–1.00)	0.61 (0.43–0.86)
1	426 (8.2)	106 (11.4)	1.56 (1.24–1.97)	1.31 (1.02–1.69)
2–3	3586 (68.9)	571 (61.3)	Reference	Reference
≥ 4	736 (14.1)	201 (21.6)	1.72 (1.43–2.05)	1.55 (1.28–1.89)
Body mass index (kg/m^2^)				
Underweight (<18.5)	90 (1.7)	16 (1.7)	1.03 (0.60–1.77)	0.79 (0.44–1.44)
Normal weight (18.5–24.9)	2802 (53.8)	482 (51.7)	Reference	Reference
Overweight (25–29.9)	1494 (28.7)	243 (26.1)	0.95 (0.80–1.12)	0.92 (0.77–1.10)
Obese (≥30)	820 (15.8)	191 (20.5)	1.35 (1.13–1.63)	1.24 (1.02–1.53)
Median BMI (Q1, Q3)	24.5 (22.1, 27.7)	24.6 (22.3, 28.7)		
Perceived stress				
No (stress scores <1)	4245 (81.5)	581 (62.3)	Reference	Reference
Yes (stress scores ≥1)	961 (18.5)	351 (37.7)	2.67 (2.30–3.10)	2.03 (1.72–2.40)
Median stress scores (Q1, Q3)	0.5 (0.2, 0.8)	0.7 (0.4, 1.2)		
Smoking status				
Never	3048 (58.5)	360 (38.6)	Reference	Reference
Ex-smoker	1469 (28.2)	316 (33.9)	1.82 (1.55–2.14)	1.73 (1.45–2.05)
Current smoker, <10 cigarettes/day	146 (2.8)	50 (5.4)	2.90 (2.07–4.07)	2.52 (1.75–3.63)
Current smoker, 10–19 cigarettes/day	178 (3.4)	47 (5.0)	2.24 (1.59–3.14)	1.80 (1.25–2.58)
Current smoker, ≥20 cigarettes/day	365 (7.0)	159 (17.1)	3.69 (2.97–4.58)	2.77 (2.19–3.51)

Data are presented as n (%), median (interquartile range) or odds ratio (OR) and 95% confidence interval (95% CI) using logistic regression models. Q1, 25th percentile; Q3, 75th percentile

^a^Adjusted model was adjusted for all the covariates listed in the table

Some similarities were evident in the characteristics of women and their time of ANM (Additional file 2: Table S2). Specifically, the risk factors for women associated with early ANM (before age 45 years) after adjusting for other factors and compared with ANM from 50 to 51 years (Table 2), included lower education levels, having income difficulties, and being a current smoker. The risk of early ANM also increased with the number of cigarettes smoked, such that heavy smokers were three times as likely (RRR 2.98, 95% CI 2.11–4.20) as having early ANM compared with those who had never smoked. Women who reported having an early menarche (at age 11 years or earlier) had an increased risk of having early ANM.

**Table 2:** Adjusted associations of socioeconomic, reproductive and lifestyle factors with age at natural menopause (*n* = 6138)

	Age at natural menopause (ANM) (years)				
	<45 RRR (95% CI)	45–49 RRR (95% CI)	50–51 RRR (95% CI)	52–53 RRR (95% CI)	≥54 RRR (95% CI)
Education level					
No formal qualifications	1.72 (1.11–2.65)	1.36 (1.03–1.81)	Reference	0.95 (0.71–1.26)	0.71 (0.54–0.92)
Less than high school/high school	1.54 (1.08–2.20)	0.96 (0.77–1.20)	Reference	0.84 (0.67–1.04)	0.71 (0.58–0.86)
Trade/certificate/diploma	1.34 (0.90–2.01)	0.95 (0.74–1.23)	Reference	0.88 (0.69–1.13)	0.76 (0.60–0.95)
University or higher	Reference	Reference	Reference	Reference	Reference
Difficulty on income management					
Easy/not bad/some difficult	Reference	Reference	Reference	Reference	Reference
Difficult/impossible	1.47 (1.08–2.02)	1.14 (0.89–1.45)	Reference	0.91 (0.71–1.18)	1.10 (0.88–1.38)
Age at menarche (years)					
≤ 11	1.54 (1.10–2.16)	1.30 (1.03–1.64)	Reference	1.09 (0.86–1.37)	1.00 (0.80–1.24)
12	1.49 (1.10–2.03)	0.95 (0.76–1.18)	Reference	0.88 (0.71–1.09)	0.92 (0.76–1.13)
13	Reference	Reference	Reference	Reference	Reference
14	1.14 (0.80–1.62)	1.13 (0.90–1.43)	Reference	0.94 (0.74–1.18)	1.09 (0.88–1.35)
≥ 15	1.34 (0.94–1.91)	1.09 (0.86–1.39)	Reference	0.98 (0.77–1.25)	1.18 (0.95–1.46)
Number of children					
0	1.28 (0.87–1.89)	1.32 (1.00–1.73)	Reference	1.00 (0.76–1.34)	0.83 (0.63–1.08)
1	1.04 (0.71–1.52)	1.16 (0.89–1.51)	Reference	0.86 (0.65–1.14)	0.86 (0.66–1.11)
2–3	Reference	Reference	Reference	Reference	Reference
≥ 4	0.99 (0.73–1.35)	0.97 (0.78–1.20)	Reference	1.04 (0.84–1.29)	0.94 (0.77–1.15)
Body mass index (kg/m^2^)					
Underweight (<18.5)	1.27 (0.57–2.83)	1.36 (0.77–2.40)	Reference	1.18 (0.65–2.14)	0.92 (0.51–1.65)
Normal weight (18.5–24.9)	Reference	Reference	Reference	Reference	Reference
Overweight (25–29.9)	1.10 (0.85–1.42)	1.06 (0.89–1.27)	Reference	1.12 (0.94–1.34)	1.34 (1.14–1.58)
Obese (≥30)	1.03 (0.75–1.41)	1.08 (0.87–1.34)	Reference	1.10 (0.88–1.37)	1.26 (1.03–1.55)
Perceived stress					
No (stress scores <1)	Reference	Reference	Reference	Reference	Reference
Yes (stress scores ≥1)	1.15 (0.88–1.50)	1.12 (0.93–1.36)	Reference	0.94 (0.77–1.14)	1.00 (0.83–1.19)
Smoking status					
Never	Reference	Reference	Reference	Reference	Reference
Ex-smoker	1.21 (0.93–1.57)	0.88 (0.74–1.05)	Reference	0.90 (0.76–1.07)	0.93 (0.80–1.09)
Current smoker, <10 cigarettes/day	1.69 (0.98–2.92)	0.84 (0.54–1.30)	Reference	0.86 (0.56–1.32)	0.73 (0.49–1.10)
Current smoker, 10–19 cigarettes/day	2.00 (1.22–3.27)	1.13 (0.77–1.67)	Reference	0.80 (0.53–1.23)	0.69 (0.46–1.03)
Current smoker, ≥20 cigarettes/day	2.98 (2.11–4.20)	1.71 (1.30–2.25)	Reference	0.82 (0.59–1.12)	0.81 (0.61–1.09)

Data are presented as relative risk ratio (RRR) and 95% confidence interval (CI) using multinomial logistic regression models, and all RRRs (95% CI) were adjusted for the covariates listed in the table

In terms of the relationship between IPV and the timing of menopause (Table 3), women who had a history of IPV were at 54% increased risk (RRR 1.54, 95% CI 1.17–2.01) of early ANM compared to the reference group (women without IPV and ANM at 50–51 years)**.** This increased risk was attenuated (RRR 1.36, 1.03–1.80) after adjustment for education level, income difficulties, age at menarche, parity, BMI, and stress (Model 6). With further adjustment for smoking status (Model 7), the risk of early menopause was further attenuated and no longer significant (RRR 1.20, 0.90–1.59). IPV was still associated with an earlier ANM (effect size: −0.39 years, 95% CI -0.69 to −0.08) in the fully adjusted model that used continuous ANM as the outcome. In the sensitivity analysis which used inverse probability weighting to account for the bias caused by the complete-case analysis, we observed similar results that the risk of early menopause was attenuated from 1.37 (1.06–1.76) to 1.19 (0.92–1.55) after adjusting for smoking status.

**Table 3:** Multivariable adjusted association between intimate partner violence and age at natural menopause (*n* = 6138)

	Age at natural menopause (ANM) (years)					
Intimate partner violence	<45 RRR (95% CI)	45–49 RRR (95% CI)	50–51 RRR (95% CI)	52–53 RRR (95% CI)	≥54 RRR (95% CI)	Continuous ANM β (95% CI)
Unadjusted model	1.54 (1.17–2.01)	1.06 (0.86–1.30)	Reference	0.87 (0.70–1.07)	0.83 (0.68–1.01)	−0.72 (−1.01 to −0.42)
Model 1: + education level	1.49 (1.14–1.95)	1.04 (0.84–1.28)	Reference	0.87 (0.70–1.08)	0.84 (0.69–1.02)	−0.66 (−0.95 to −0.36)
Model 2: Model 1 + income difficulties	1.39 (1.06–1.83)	1.01 (0.82–1.24)	Reference	0.88 (0.71–1.09)	0.82 (0.67–1.00)	−0.60 (−0.89 to −0.30)
Model 3: Model 2 + menarche	1.38 (1.05–1.82)	1.00 (0.81–1.23)	Reference	0.87 (0.70–1.09)	0.82 (0.67–1.00)	−0.60 (−0.89 to −0.30)
Model 4: Model 3 + parity	1.39 (1.05–1.83)	1.00 (0.81–1.24)	Reference	0.87 (0.70–1.09)	0.82 (0.67–1.00)	−0.62 (−0.92 to −0.32)
Model 5: Model 4 + BMI	1.39 (1.05–1.83)	1.00 (0.81–1.24)	Reference	0.87 (0.70–1.09)	0.82 (0.67–1.00)	−0.62 (−0.92 to −0.33)
Model 6: Model 5 + stress status	1.36 (1.03–1.80)	0.98 (0.80–1.22)	Reference	0.88 (0.71–1.10)	0.82 (0.67–1.00)	−0.60 (−0.90 to −0.29)
Model 7: Model 6 + smoking status	1.20 (0.90–1.59)	0.95 (0.76–1.17)	Reference	0.91 (0.73–1.13)	0.84 (0.68–1.03)	−0.39 (−0.69 to −0.08)

Multinominal logistic regression models were used to estimate relative risk ratio (RRR) and 95% confidence intervals (CI) for the categorical ANM. Linear regression models were used to estimate β (95% CI) for the continuous ANM

The results of the multivariable mediation analysis are presented in Table 4. More than one-third (36.7%) of the association of IPV with increased risk of early menopause (ANM <45 years vs. ≥45 years) (OR 1.49, 95% CI 1.16–1.90) was mediated through the number of cigarettes smoked per day (five categories), after adjusting for education level, income difficulties, and age at menarche. The experience of IPV was also associated with having earlier ANM (−0.60 years, 95% CI -0.89 to −0.30) after full adjustment. As above, the number of cigarettes smoked per day accounted for 37.0% of the effect associated between IPV and the timing of ANM. For both early ANM and the timing of ANM, a similar proportion was mediated through smoking when smoking status was dichotomised as current and non-current smoking.

**Table 4:** Natural direct and indirect effects of intimate partner violence on the age at natural menopause and the proportion mediated through smoking (*n* = 6138)^a^

	OR^NDE^ (95% CI)	OR^NIE^ (95% CI)	OR^TE^ (95% CI)	Proportion mediated by smoking (%)
Early menopause (ANM <45 vs. ≥45 years)				
Mediator: smoking				
Smoking status (5 categories)	1.31 (1.02–1.68)	1.14 (1.09–1.18)	1.49 (1.16–1.90)	36.7
Current vs. non-current smoking (2 categories)	1.34 (1.04–1.72)	1.13 (1.08–1.18)	1.51 (1.18–1.93)	33.7
	β^NDE^ (95% CI)	β^NIE^ (95% CI)	β^TE^ (95% CI)	Proportion mediated by smoking (%)
Continuous ANM				
Mediator: smoking				
Smoking status (5 categories)	−0.38 (−0.68 to −0.08)	−0.22 (−0.28 to −0.16)	−0.60 (−0.89 to −0.30)	37.0
Current vs. non-current smoking (2 categories)	−0.41 (−0.71 to −0.11)	−0.18 (−0.23 to −0.12)	−0.59 (−0.94 to −0.24)	29.8

*ANM* age at natural menopause, *NDE* natural direct effect, *NIR* natural indirect effect, *TE* total effect

^a^All estimates were adjusted for education level, income difficulties, and age at menarche. The mediation analysis was performed by fitting a logistic regression model for the binary outcome (ANM <45 vs. ≥45 years) and a linear regression model for the continuous ANM and fitting a linear or logistic regression model for the ordinal or binary mediator. The proportion mediated was calculated as [(OR^NDE^ (OR^NIE^ – 1)] / [OR^NDE^ × OR^NIE^ –1] × 100% for the binary outcome or (β^NIE^/β^TE^) × 100% for the continuous outcome

## Discussion

Using data from a large population-based cohort, this study underscores that women with a history of IPV in midlife are at increased risk of obesity and are more likely to report having four or more children, being stressed and be current smokers, especially heavy smoking (20 or more cigarettes per day). To our knowledge, this is the first study to show that IPV is also associated with increased risk of early ANM (before age 45 years) after adjusting for other risk factors. This relationship was attenuated, however, after accounting for smoking, which is an established risk factor for early ANM. Findings from the mediation analyses using a counterfactual framework confirmed that a considerable part of the link between IPV and earlier ANM was mediated via current smoking status. Specifically, the number of cigarettes smoked per day explained more than one third (36.7%) of the overall relationship of IPV with early ANM after adjusting for education level, income difficulties, and age at menarche. These findings are consistent with those from previous studies that have shown the strong links between IPV and higher rates of cigarette smoking [8–10] and between smoking and earlier ANM [18].

The association between IPV and tobacco use is well known and has repeatedly been reported in the literature [8–10]. While causality has not been demonstrated, the direct link approach such as that used in the current analysis is justified. A systematic review of IPV and tobacco use literature found the only factor that influenced the association between IPV and tobacco was pregnancy [31], a factor that is not relevant to the current analysis. The review authors propose that nicotine acts to both alleviate symptoms of depression that are caused by IPV and the symptoms of anxiety that accompany living with a violent partner [31]. More research is needed to allow for the development of effective smoking cessation interventions for women who have experienced IPV. It is possible that smoking cessation programs designed for those experiencing mental health disorders might be more effective than standard smoking cessation programs, given the much lower quit rates found among those with mental health problems [32]. It should be noted that while certain characteristics seem to cluster together (for example, cigarrette smoking, low education level, income difficulties, being stressed and being exposed to violence), cigarette smoking, by far, has been shown to be consistently linked with age at menopause. We also found that around 5% of the association between IPV and risk of early menopause was mediated through stress (without considering smoking status), but no significant joint effect between smoking status and stress status was observed (data not shown). Therefore, health promotion effort should be placed on smoking cessation programs.

IPV has been linked with sexually transmitted and reproductive disorders (e.g. cervical cancer, vaginal discharge) [2, 6, 7] but this is the first study to demonstrate a clear relationship with early reproductive ageing, which in turn has a known impact on cardiovascular disease [16]. Links have previously been shown between IPV and cardiovascular disease and metabolic syndrome disorder, with a strong focus on health behaviours (e.g. smoking, abdominal obesity) and mental distress as mechanisms that connect IPV with cardiovascular risk [33]. The current study adds to knowledge in this regard by highlighting the importance of reproductive ageing. Future research will need to take into account the multiple social and biological pathways through which IPV acts to influence health and well-being. Early life experience of violence may also affect ovarian function and reproductive ageing via stress response dysregulation [34, 35]. One cohort study found that women who experienced childhood or adolescent violence had more extreme levels of ovarian hormones during perimenopause, suggesting that early experience of violence may lead to neuroendocrine disruption, thereby affecting ovarian function [35]. However, this study found conflicting findings that early life violence was associated with delayed (rather than early) onset of perimenopause (measured by menstrual changes) [34]. Our previous cross-sectional analysis using baseline data found that IPV was associated with surgical menopause but not with postmenopause and perimenopause after adjustment for demographic and health behaviour variables [7]. A number of reasons may explain the conflicting results including different outcomes (hormones and menstrual cycle/status), different forms of violence (physical, emotional, sexual), and different analytical approaches. Hence, more studies are needed to prove the link between the experience of all forms of violence and reproductive health.

The strengths of our study included large sample size and nationally representative study population, which improves the generalizability of our findings to other middle-aged women. Our study was strengthened by its prospective nature, particularly with respect to longitudinal data on the timing of menopause. This meant that reverse causation could be ruled out for the relationships observed between IPV at baseline and ANM. The extensive survey data collected from the women has also allowed adjustment for a wide range of confounders, including known risk factors for IPV and earlier ANM. However, there were some limitations that should be acknowledged. First, all the data were self-reported, which may have led to some under-reporting of IPV. It was also the case that women who were excluded from the analyses due to incomplete data were more likely to have a history of IPV, be a current smoker, and have an early ANM compared to those who were included. If this potential underestimation of the prevalence of IPV, current smoking, and early menopause were included, it seems likely that the observed associations would be strengthened. Second, since the study is limited to Australian women, these findings should be replicated in other populations. Further research is also needed to investigate potential mechanisms for the relationship between IPV and ANM that is not explained by smoking and other risk factors in this study.

## Conclusions

Women who had a prior history of IPV were at increased risk of early menopause (<45 years), with this relationship substantially mediated through smoking. Our findings suggest that as part of addressing the issue of IPV and its subsequent consequences for women, smoking cessation interventions tailored for women who have lived with IPV will partly mitigate the links with earlier menopause, which is an established risk factor for a range of adverse health outcomes in later life in addition to the effects of smoking.

## Additional files

Additional file 1: Table S1. Characteristics of excluded and included women. (DOCX 14 kb)

Additional file 2: Table S2. Characteristics of women according to age at natural menopause (*n* = 6138). (DOCX 15 kb)

## Abbreviations

ALSWH: Australian Longitudinal Study on Women’s Health
ANM: Age at natural menopause
BMI: Body mass index
CI: Confidence interval
IPV: Intimate partner violence
NDR: Natural direct effect
NIR: Natural indirect effect
OR: Odds ratio
RRR: Relative risk ratio
TE: Total effect
WHO: World Health Organization
