# Supplementary material for: The role of smoking in the relationship between intimate partner violence and age at natural menopause: a mediation analysis
Source: Womens Midlife Health. 2018 Jan 15;4:1. doi: 10.1186/s40695-017-0031-9 (PMC6297990; doi:10.1186/s40695-017-0031-9)
Supplement: Supplementary file 1 — Characteristics of excluded and included women. (DOCX 14 kb) [file 40695_2017_31_MOESM1_ESM.docx]

**Additional file 1**

**Table S1** Characteristics of excluded and included women

|  | Excluded  (n=1,497) | Included  (n=6,138) | P-value |
| --- | --- | --- | --- |
| Area of residency |  |  |  |
| Urban | 568 (37.9) | 2220 (36.2) | 0.2011 |
| Rural/remote | 929 (62.1) | 3918 (63.8) |  |
| Education level |  |  |  |
| No formal qualifications | 287 (20.1) | 834 (13.6) | <0.0001 |
| Less than high school/high school | 689 (48.2) | 2965 (48.3) |  |
| Trade/certificate/diploma | 249 (17.4) | 1273 (20.7) |  |
| University or higher | 204 (14.3) | 1066 (17.4) |  |
| Difficulty on income management |  |  | 0.0002 |
| Easy/not bad/some difficult | 1223 (84.1) | 5386 (87.7) |  |
| Difficult/impossible | 231 (15.9) | 752 (12.3) |  |
| Marital status |  |  |  |
| Married/de facto | 1142 (77.7) | 5154 (84.1) | <0.0001 |
| Separated/divorced | 209 (14.2) | 671 (10.9) |  |
| Widowed | 33 (2.2) | 115 (1.9) |  |
| Single | 85 (5.8) | 191 (3.1) |  |
| Age at menarche (years) |  |  |  |
| ≤11 | 119 (17.6) | 1067 (17.4) | 0.9187 |
| 12 | 145 (21.5) | 1287 (21.0) |  |
| 13 | 192 (28.4) | 1784 (29.1) |  |
| 14 | 121 (17.9) | 1043 (17.0) |  |
| ≥15 | 98 (14.5) | 957 (15.6) |  |
| Number of children |  |  |  |
| 0 | 96 (7.4) | 512 (8.3) | 0.0256 |
| 1 | 100 (7.7) | 532 (8.7) |  |
| 2-3 | 863 (66.5) | 4157 (67.7) |  |
| ≥4 | 239 (18.4) | 937 (15.3) |  |
| Body mass index (kg/m^2^) |  |  |  |
| Underweight (<18.5) | 27 (2.2) | 106 (1.7) | 0.5826 |
| Normal weight (18.5-24.9) | 656 (52.6) | 3284 (53.5) |  |
| Overweight (25-29.9) | 366 (29.4) | 1737 (28.3) |  |
| Obese (≥30) | 197 (15.8) | 1011 (16.5) |  |
| Median BMI (Q1, Q3) | 24.5 (22.3, 27.8) | 24.5 (22.2, 27.9) |  |
| Perceived stress |  |  |  |
| No (stress scores <1) | 1053 (72.1) | 4826 (78.6) | <0.0001 |
| Yes (stress scores ≥1) | 408 (27.9) | 1312 (21.4) |  |
| Median stress scores (Q1, Q3) | 0.6 (0.3, 1.0) | 0.5 (0.3, 0.9) |  |
| Smoking status |  |  |  |
| Never | 655 (52.0) | 3408 (55.5) | <0.0001 |
| Ex-smoker | 323 (25.7) | 1785 (29.1) |  |
| Current smoker, <10 cigarettes/day | 58 (4.6) | 196 (3.2) |  |
| Current smoker, 10-19 cigarettes/day | 65 (5.2) | 225 (3.7) |  |
| Current smoker, ≥20 cigarettes/day | 158 (12.5) | 524 (8.5) |  |
| Intimate partner violence |  |  |  |
| No | 1149 (79.1) | 5206 (84.8) | <0.0001 |
| Yes | 304 (20.9) | 932 (15.2) |  |
| Age at natural menopause (years) |  |  |  |
| <45 | 118 (7.9) | 429 (7.0) | <0.0001 |
| 45-49 | 378 (25.3) | 1263 (20.6) |  |
| 50-51 | 390 (26.1) | 1434 (23.4) |  |
| 52-53 | 246 (16.4) | 1245 (20.3) |  |
| ≥54 | 365 (24.4) | 1767 (28.8) |  |
| Median ANM (Q1,Q3) | 50.0 (48.0, 53.0) | 51.0 (49.0, 54.0) |  |

Data are presented as n (%) or median (interquartile range). BMI, body mass index; ANM, age at natural menopause; Q1, 25^th^ percentile; Q3, 75^th^ percentile.
