# Supplementary material for: The role of smoking in the relationship between intimate partner violence and age at natural menopause: a mediation analysis
Source: Womens Midlife Health. 2018 Jan 15;4:1. doi: 10.1186/s40695-017-0031-9 (PMC6297990; doi:10.1186/s40695-017-0031-9)
Supplement: Supplementary file 2 — Characteristics of women according to age at natural menopause (n = 6138). (DOCX 15 kb) [file 40695_2017_31_MOESM2_ESM.docx]

**Additional file 2**

**Table S2** Characteristics of women according to age at natural menopause (n=6,138)

|  | Age at natural menopause (ANM) (years) | | | | |  |
| --- | --- | --- | --- | --- | --- | --- |
|  | <45  (n=429, 7.0%) | 45-49  (n=1263, 20.6%) | 50-51  (n=1434, 23.4%) | 52-53  (n=1245, 20.3%) | ≥54  (n=1767, 28.8%) | P-value |
| Education level |  |  |  |  |  |  |
| No formal qualifications | 71 (16.6) | 218 (17.3) | 176 (12.3) | 163 (13.1) | 206 (11.7) | <0.0001 |
| Less than high school/high school | 229 (53.4) | 597 (47.3) | 719 (50.1) | 592 (47.6) | 828 (46.9) |  |
| Trade/certificate/diploma | 83 (19.3) | 248 (19.6) | 307 (21.4) | 264 (21.2) | 371 (21.0) |  |
| University or higher | 46 (10.7) | 200 (15.8) | 232 (16.2) | 226 (18.2) | 362 (20.5) |  |
| Difficulty on income management |  |  |  |  |  | 0.0002 |
| Easy/not bad/some difficult | 352 (82.1) | 1089 (86.2) | 1273 (88.8) | 1119 (89.9) | 1553 (87.9) |  |
| Difficult/impossible | 77 (17.9) | 174 (13.8) | 161 (11.2) | 126 (10.1) | 214 (12.1) |  |
| Age at menarche (years) |  |  |  |  |  |  |
| ≤11 | 85 (19.8) | 248 (19.6) | 228 (15.9) | 223 (17.9) | 283 (16.0) | 0.0242 |
| 12 | 111 (25.9) | 249 (19.7) | 318 (22.2) | 250 (20.1) | 359 (20.3) |  |
| 13 | 100 (23.3) | 351 (27.8) | 431 (30.1) | 385 (30.9) | 517 (29.3) |  |
| 14 | 64 (14.9) | 223 (17.7) | 241 (16.8) | 201 (16.1) | 314 (17.8) |  |
| ≥15 | 69 (16.1) | 192 (15.2) | 216 (15.1) | 186 (14.9) | 294 (16.6) |  |
| Number of children |  |  |  |  |  |  |
| 0 | 41 ( 9.6) | 126 (10.0) | 115 ( 8.0) | 103 ( 8.3) | 127 ( 7.2) | 0.1197 |
| 1 | 41 ( 9.6) | 128 (10.1) | 128 ( 8.9) | 95 ( 7.6) | 140 ( 7.9) |  |
| 2-3 | 279 (65.0) | 821 (65.0) | 973 (67.9) | 849 (68.2) | 1235 (69.9) |  |
| ≥4 | 68 (15.9) | 188 (14.9) | 218 (15.2) | 198 (15.9) | 265 (15.0) |  |
| Body mass index (kg/m^2^) |  |  |  |  |  |  |
| Underweight (<18.5) | 9 ( 2.1) | 28 ( 2.2) | 23 ( 1.6) | 22 ( 1.8) | 24 ( 1.4) | 0.1823 |
| Normal weight (18.5-24.9) | 225 (52.4) | 675 (53.4) | 811 (56.6) | 671 (53.9) | 902 (51.0) |  |
| Overweight (25-29.9) | 121 (28.2) | 343 (27.2) | 378 (26.4) | 351 (28.2) | 544 (30.8) |  |
| Obese (≥30) | 74 (17.2) | 217 (17.2) | 222 (15.5) | 201 (16.1) | 297 (16.8) |  |
| Median BMI (Q1, Q3) | 24.5 (22.4, 28.2) | 24.5 (22.0, 28.1) | 24.2 (22.0, 27.6) | 24.3 (22.0, 27.6) | 24.8 (22.3, 28.2) |  |
| Perceived stress |  |  |  |  |  |  |
| No (stress scores <1) | 320 (74.6) | 969 (76.7) | 1138 (79.4) | 1004 (80.6) | 1395 (78.9) | 0.0334 |
| Yes (stress scores ≥1) | 109 (25.4) | 294 (23.3) | 296 (20.6) | 241 (19.4) | 372 (21.1) |  |
| Median stress scores (Q1, Q3) | 0.6 (0.3, 1.0) | 0.5 (0.3, 0.9) | 0.5 (0.3, 0.9) | 0.5 (0.2, 0.8) | 0.5 (0.3, 0.9) |  |
| Smoking status |  |  |  |  |  |  |
| Never | 181 (42.2) | 673 (53.3) | 791 (55.2) | 729 (58.6) | 1034 (58.5) | <0.0001 |
| Ex-smoker | 123 (28.7) | 335 (26.5) | 435 (30.3) | 360 (28.9) | 532 (30.1) |  |
| Current smoker, <10 cigarettes/day | 20 ( 4.7) | 37 ( 2.9) | 51 ( 3.6) | 40 ( 3.2) | 48 ( 2.7) |  |
| Current smoker, 10-19 cigarettes/day | 27 ( 6.3) | 55 ( 4.4) | 55 ( 3.8) | 40 ( 3.2) | 48 ( 2.7) |  |
| Current smoker, ≥20 cigarettes/day | 78 (18.2) | 163 (12.9) | 102 ( 7.1) | 76 ( 6.1) | 105 ( 5.9) |  |
| Intimate partner violence |  |  |  |  |  |  |
| No | 334 (77.9) | 1056 (83.6) | 1210 (84.4) | 1073 (86.2) | 1533 (86.8) | <0.0001 |
| Yes | 95 (22.1) | 207 (16.4) | 224 (15.6) | 172 (13.8) | 234 (13.2) |  |

Data are presented as n (%) or median (interquartile range). BMI, body mass index; ANM, age at natural menopause; Q1, 25^th^ percentile; Q3, 75^th^ percentile.
